# Thoracic load carriage impairs the acute physiological response to hypoxia in healthy males

**DOI:** 10.14814/phy2.70197

**Published:** 2025-01-08

**Authors:** Daniel A. Baur, Caroline M. Lassalle, Stephanie P. Kurti

**Affiliations:** ^1^ Department of Human Performance and Wellness Virginia Military Institute Lexington Virginia USA; ^2^ Department of Kinesiology James Madison University Harrisonburg Virginia USA

**Keywords:** altitude, expiratory flow limitation, hemodynamics, near‐infrared spectroscopy, respiratory muscle fatigue, ventilation

## Abstract

To assess the impact of thoracic load carriage on the physiological response to exercise in hypoxia. Healthy males (*n* = 12) completed 3 trials consisting of 45 min walking in the following conditions: (1) unloaded normoxia (UN; F_I_O_2_:20.93%); (2) unloaded hypoxia (UH; F_I_O_2_:~13.0%); and (3) loaded hypoxia (LH; 29.5 kg; F_I_O_2_:~13.0%). Intensity was matched for absolute VO_2_ (2.0 ± 0.2 L·min^−1^) across conditions and relative VO_2_ (64.0 ± 2.6 %VO_2max_) across hypoxic conditions. With LH versus UH, there were increases in breathing frequency (5–11 breaths·min^−1^; *p* < 0.05) and decreases in tidal volume (10%–18%; *p* < 0.05) throughout exercise due to reductions in end inspiratory lung volumes (*p* < 0.05). Consequently, deadspace (11%–23%; *p* < 0.05) and minute ventilation (7%–11%; *p* < 0.05) were increased starting at 20 and 30 min, respectively. In addition, LH increased perceived exertion/dyspnea and induced inspiratory (~12%; *p* < 0.05 vs. UN) and expiratory (~10%; *p* < 0.05 vs. pre‐exercise) respiratory muscle fatigue. Expiratory flow limitation was present in 50% of subjects during LH. Cardiac output and muscle oxygenation were maintained during LH despite reduced stroke volume (6%–8%; *p* < 0.05). Finally, cerebral oxygenated/total hemoglobin were elevated in the LH condition versus UH starting at 15 min (*p* < 0.05). Thoracic load carriage increases physiological strain and interferes with the compensatory response to hypoxic exposure.

## 1 INTRODUCTION

Various populations including hikers, military personnel, and wildland firefighters operate or recreate in high altitude (>3000 m) mountainous environments (Rodway & Muza, 2011; Ruby et al., 2023). In high altitudes, the partial pressure of oxygen is reduced leading to arterial hypoxemia and tissue hypoxia (Young & Reeves, 2002). This necessitates several compensatory physiological responses, such as increases in cardiac output [Q], minute ventilation [V_E_], and arterial blood pressure. These responses, while adequate for maintaining submaximal tissue oxygenation, limit aerobic power/endurance (Young & Reeves, 2002).

Importantly, operating in these typically remote and inaccessible environments often requires thoracic load carriage for the transport of essential equipment and supplies (Faghy et al., 2022). While necessary, thoracic load carriage impairs cardiopulmonary function and increases perceived exertion/dyspnea (Faghy et al., 2022). Taken together, it seems likely that thoracic load carriage may add to the physiological strain or interfere with the compensatory response to hypoxia in ways likely to further compromise health and performance. However, the physiological effects of hypoxic load carriage are yet to be fully elucidated.

Specifically, it is unknown whether the load carriage‐induced impairments in ventilatory function are exacerbated in hypoxia. Worsened cardiopulmonary responses in hypoxia seem likely for two reasons. First, thoracic load carriage results in a shallow breathing pattern, which increases deadspace ventilation and V_E_ over time (Phillips, Stickland, & Petersen, 2016b, 2016c). This increase in respiratory work has been associated with respiratory muscle fatigue in normoxia (Phillips, Stickland, & Petersen, 2016b, 2016c). In hypoxia, it is conceivable that these effects may be worsened given the reported greater diaphragm and abdominal muscle fatiguability during exercise and generally higher ventilatory demands (Verges et al., 2010). Second, normoxic load carriage has been reported by some (Armstrong et al., 2019; Dominelli et al., 2012), but not others (Shei et al., 2018), to result in increased expiratory flow limitation (EFL), but mostly with very heavy loads (i.e., 30–50 kg) and high work rates (i.e., >3.0 L·min^−1^) that result in V_E_ > ~70 L·min^−1^. Hence, EFL may be more common with hypoxic load carriage where these ventilatory demands occur at lower work rates. If true, maintenance of V_E_ with EFL would require increases in operating lung volumes requiring breathing patterns that fall on less compliant portions of the pressure‐volume curve thereby increasing the work of breathing (W_b_; joules·breath^−1^) and hastening the development of respiratory muscle fatigue (Stark‐Leyva et al., 2004). Two studies have assessed the ventilatory effects of hypoxic load carriage and observed seemingly impaired ventilatory function including reduced or increased V_T_ and f_B_, respectively, and respiratory muscle fatigue (Baur et al., 2023; Hinde et al., 2018). However, these studies employed either relatively low‐intensity exercise at self‐selected walking speeds (i.e., not matched for oxygen consumption [VO_2_]; (Hinde et al., 2018)), or did not have an unloaded hypoxic comparison condition (Baur et al., 2023). Additionally, neither study assessed EFL. Thus, it is impossible to determine from these studies whether or to what degree load carriage impairs the ventilatory response to hypoxia.

Importantly, potential load carriage‐induced ventilatory impairments in hypoxia have important implications for cardiovascular function and locomotor muscle oxygenation. For instance, EFL reduces Q (i.e., due to a reduction in SV) and arterial oxygen saturation likely as a result of increased expiratory pressures that reduce venous return and pulmonary capillary blood volume (Aliverti et al., 2005). Collectively, these effects may additively induce or exacerbate respiratory muscle fatigue and cause a metaboreflex. Prior work has established that the respiratory muscle metaboreflex can effectively “steal” blood flow from locomotor muscle when Q is limiting to performance (>85% VO_2max_; (Hinde et al., 2018)). While this has not been observed with increased respiratory muscle work during lower‐intensity (40% VO_2peak_) exercise in normoxia (Katayama et al., 2019), effects may be more apparent in hypoxia where the respiratory muscle metaboreflex is exacerbated and locomotor muscle fatigue is more clearly influenced by *W*_b_ (Amann, Pegelow, et al., 2007; Katayama et al., 2013a). Nevertheless, only one study has investigated the effects of normoxic or hypoxic load carriage on Q and muscle oxygenation (Baur et al., 2023). Interestingly, this study reported reductions in SV during VO_2_‐matched normoxic load carriage and a reduction in muscle oxygenation with hypoxic load carriage matched for walking speed with unloaded normoxic exercise. However, there were no measurements of EFL, metaboreflex variables (e.g., arterial blood pressure), or respiratory muscle fatigue. Moreover, the lack of an unloaded hypoxic comparison condition prevents isolation of load carriage‐specific effects independent of load/speed‐ and F_I_O_2_‐mediated differences in energy expenditure and ventilatory demand. Therefore, more research is needed to determine whether hypoxic load carriage influences cardiac output or locomotor muscle oxygenation.

Finally, hypoxic load carriage may influence cerebral blood flow and oxygenation in ways that may compromise health and performance. Indeed, prior research has indicated that cerebral oxygenation/blood flow can be reduced in hypoxic environments during exercise due to hypoxemia and hyperventilation‐induced hypocapnia (Fan & Kayser, 2016), and that these effects may mediate performance independent of other factors that contribute to fatigue (e.g., muscle blood flow/oxygenation; (Amann, Romer, et al., 2007)). Given the independent effects of hypoxia and load carriage, it is conceivable that their combination may exacerbate cerebral oxygenation/blood flow deficiencies. Surprisingly, Baur et al. (2023) recently observed evidence for a load carriage‐mediated effect on cerebral oxygen kinetics suggesting *increased* prefrontal cortex blood flow. Specifically, normoxic and hypoxic load carriage increased oxygenated hemoglobin (O_2_HHb) and total hemoglobin (tHHb), respectively, compared to VO_2_‐matched unloaded normoxic exercise. This counterintuitive finding requires more research to confirm these effects in hypoxia‐matched conditions, particularly given the ubiquity of load carriage in high altitudes and the fact that increases in cerebral blood flow/perfusion have been implicated in the pathophysiology of acute mountain sickness (AMS) and high‐altitude cerebral edema (HACE; (Taylor 2011)).

Therefore, the purpose of this study was to investigate the physiological effects of prolonged hypoxic load carriage.

## 2 MATERIALS AND METHODS

### 2.1 Study design and ethical approval

This was a randomized, counterbalanced, single‐blinded, and crossover designed study to investigate the physiological effects of hypoxic thoracic load carriage. For each subject, the study required ~2 weeks and required six laboratory visits consisting of: (1–3) baseline and familiarization and (4–6) experimental trials. Experimental trials consisted of three conditions: unloaded normoxia (UN; F_I_O_2_: 20.93%), unloaded hypoxia (UH; F_I_O_2_: ~13.0%), and loaded hypoxia (LH; F_I_O_2_: ~13.0%). For LH, subjects carried a 29.5 kg load consisting of a large Modular Lightweight Load‐Carrying Equipment pack containing cotton packing material, a ~ 20 kg sandbag, and weight plates. The pack was fitted for each subject with chest and hips straps fastened. The mass was chosen because it corresponds with the typical fighting load carried by combat soldiers (Dean, 2004). For the hypoxic conditions, normobaric hypoxic generators (Everest II, Hypoxico, Gardiner, NY, USA) were employed to simulate an altitude of ~3650 m (i.e., equivalent to ~13.0% when accounting for laboratory elevation [~325 m], barometric pressure (~738 mmHg), and 47 mmHg water vapor pressure Conkin, 2011). For all visits, subjects wore exercise clothing and running shoes. All visits were separated by ≥48 h.

During the initial visit, written informed consent was obtained following a full description of all study requirements. The study conforms to recognized national ethical standards and the Declaration of Helsinki. Subjects were excluded from participation given a diagnosis for the sickle cell trait, which can increase the risks for complications during hypoxic exercise. In the case that a subject was unaware of his sickle cell status, blood testing at the Post Infirmary was conducted for confirmation. Additionally, subjects with recent altitude exposure (i.e., had traveled to ≥1500 m within the last 3 months) were excluded from the study. All study protocols were approved by the Virginia Military Institute Institutional Review Board.

### 2.2 Subjects

Healthy recreationally active (i.e., participating in aerobic or resistance exercise ≥3 times per week) males (*n* = 14; age = 20 ± 1 years; height = 180.1 ± 4.0 cm; mass = 81.8 ± 5.6 kg; body fat (%) = 16.9 ± 5.5%) with varying amounts load carriage experience from Virginia Military Institute were recruited for this study. One subject was removed from the analysis due to noncompliance with control procedures. Additionally, one subject was unable to complete the experimental protocol in the LH condition. Therefore, results are presented as *n* = 12. Due to technical difficulties with hemodynamic and maximal inspiratory/expiratory pressure (MIP and MEP) measurements, data are presented as *n* = 11 and *n* = 10 for those variables, respectively.

### 2.3 Baseline testing

The first three laboratory visits consisted of baseline testing and familiarization. Height, weight, and body composition were assessed on the first visit with a stadiometer, electronic scale, and air displacement plethysmography, respectively (BodPod, Cosmed, Inc., Rome, Italy). Height and weight were obtained in all subsequent visits, following the same procedures. After the measurements for baseline characteristics, subsequent testing was conducted randomly in either in normoxia (F_I_O_2_ = 20.93%) or hypoxia (F_I_O_2_ = ~13.0%). First, subjects performed pulmonary function testing consistent with ATS guidelines (Graham et al., 2019) via an automated metabolic and spirometry analysis system calibrated according to manufacturer's instructions (Metalyzer 3B, Cortex, Leipzig, Germany). Thereafter, subjects completed an unloaded (i.e., in normoxia) or loaded (i.e., in hypoxia) incremental exercise test to establish VO_2_/speed relationships to permit later intensity matching based on relative VO_2max_ (i.e., 65 %VO_2max_) as described previously (Baur et al., 2023). The test consisted of 4–5 × 3‐min stages at 8% gradient in which walking speeds increased from ~2.4 to 6.5 km·h^−1^. Following 5 min seated rest, subjects then completed an additional unloaded incremental test to determine aerobic capacity (VO_2max_). The tests consisted of 1‐min stages commencing at 9.7 km·h^−1^ and 1% gradient. Next, the speed was increased by 1.6 km·h^−1^ until 12.9 km·h^−1^ was reached; thereafter, speed was held constant and the gradient was increased 1% each min until volitional exhaustion. Ratings of perceived exertion (RPE) were obtained within the last 30 s of each stage, and blood lactate (Lactate Plus, Nova Biomedical, Waltham, MA, USA) was measured ~3 min after completion of the test. VO_2max_ criteria included meeting two of the following: plateau of VO_2_ in the last stage of exercise, respiratory exchange ratio (RER) ≥1.15, heart rate (HR) within 10 beats per minute of age‐predicted maximum (220‐age), RPE ≥9 (out of 10), and blood lactate ≥8 mmol⋅L^−1^ (Howley et al., 1995). For the third laboratory visit, subjects were familiarized with experimental trial procedures. Specifically, subjects completed MIP and MEP maneuvers until two maximal values within 5 cmH_2_O were obtained. After this, subjects completed 2 × 15 min bouts (i.e., unloaded followed by loaded) of steady state walking at target speeds for experimental trials. Within the first 5 min of each bout, speed was adjusted and finalized to align with the target intensity (i.e., 65% of hypoxic VO_2max_; 2.0 ± 0.2 L·min^−1^). In the last 1 min of each 5‐min segment, subjects completed multiple inspiratory capacity (IC) maneuvers, which were visually assessed by investigators to confirm acceptability.

### 2.4 Experimental trials

**FIGURE 1:**
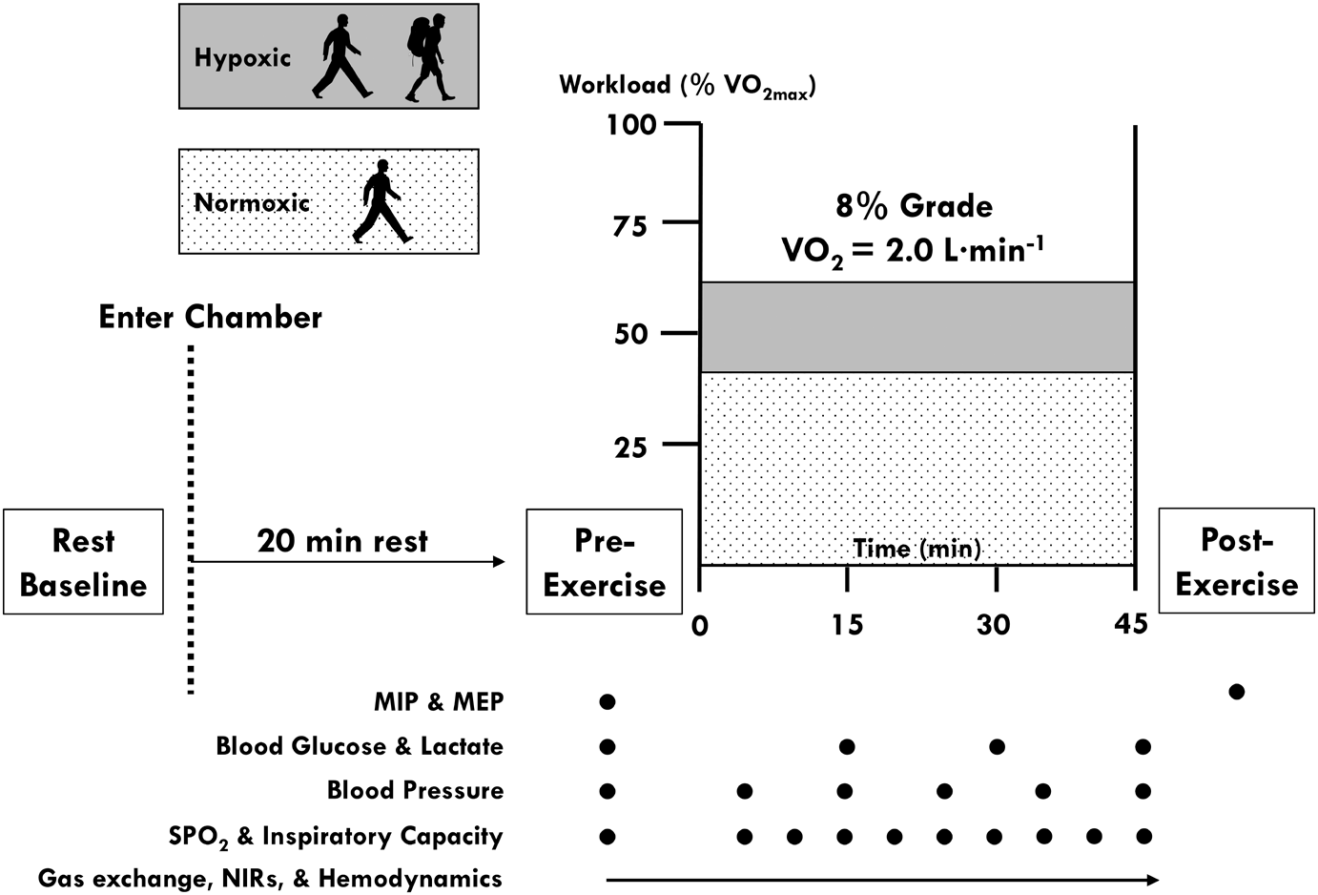
Experimental design and protocol. MEP, maximal expiratory pressure; MIP, maximal inspiratory pressure; NIRs, near‐infrared spectroscopy.

The experimental trial protocol is presented in Figure 1. Subjects arrived at the laboratory following ≥4 h fast. Upon arrival, subjects were weighed and prepared for instrumentation (e.g., placement of electrodes, blood pressure [BP] cuff, etc.). Next, subjects entered the enclosed chamber and rested for 10 min while seated. Thereafter, resting baseline measurements were collected for 5 min. Subjects then stood (and were fitted with the pack in the LH condition) and completed MIP and MEP testing before beginning the exercise protocol. Specifically, subjects completed 45 min of constant treadmill walking (~2–4 mph; 8% grade) at individually customized speeds to elicit a consistent absolute VO_2_ (2.0 ± 0.2 L·min^−1^) across all conditions and relative VO_2_ (64 ± 2.6 %VO_2max_) across hypoxic conditions. Post‐exercise MIP and MEP were assessed immediately following removal of the gas exchange mask (i.e., generally within 1–2 min, but no later than 4 min post‐exercise) of completing the exercise protocol.

### 2.5 Cardiovascular responses

Impedance cardiography (Physioflow Enduro, Manatec Biomedical, Poissy, France) was used to assess cardiac output (Q), stroke volume (SV), and heart rate (HR). These measurements were collected by the placement of six electrodes on the chest and neck, per the manufacturer's instructions. Alcohol swabs and abrasive gel (Nuprep, Weaver and Company, Aurora, CO, USA) were used to clean the surface of the skin prior to application of the electrodes. Measurements were taken at rest and continuously throughout the trial. The last 30 s of each 5‐min segment was averaged for analysis. Additionally, an automated blood pressure system (Tango, SunTech Medical, Morrisville, NC, USA) was used to assess resting and exercise arterial blood pressures (i.e., systolic [SBP], diastolic [DBP], and mean arterial blood pressures [MAP]). MAP was calculated as follows:

$$\text{Resting}\;\mathrm{MAP}=\mathrm{DBP}+1/3^{*}\;\left(\mathrm{SBP}–\mathrm{DBP}\right)$$

$$\text{Exercise}\;\mathrm{MAP}=\mathrm{DBP}+2/3^{*}\;\left(\mathrm{SBP}–\mathrm{DBP}\right)$$

For exercise blood pressure, a 4‐lead ECG (1200 W, Norav Medical, Delray Beach, FL, USA) was utilized per manufacturer's instructions. Measurements were conducted at rest and every 10 min during exercise. Hemoglobin saturation (SpO_2_) was also measured continuously with a pulse oximeter (Wrist Ox_2_ Model 3150, Nonin, Plymouth, MN, USA), and 30‐s averages were obtained every 5 min.

### 2.6 Oxygen kinetics

Near‐infrared spectroscopy (Octamon and Portamon, Artinis Medical Systems, The Netherlands) was used to assess skeletal muscle and cerebral oxygenated (O_2_HHb), deoxygenated (HHb), and total hemoglobin (tHHb) as previously described (Baur et al., 2023). For muscle, a small area on the right lateral gastrocnemius was shaved before placing the device, which was then wrapped with a black‐colored elastic bandage to prevent entry of light. For cerebral oxygen kinetics, a device was worn over the subject's forehead 1 cm above the eyebrows. For muscle, data were collected at 10 Hz and with a differential pathlength factor of 4.94 (Duncan et al., 1995). For cerebral measures, the differential pathlength factors was set based upon the subject's age (Duncan et al., 1995). Tissue oxygenation was assessed continuously, and 30‐s averages were taken at the end of every 5‐min segment. All variables were analyzed as the change relative to baseline values taken during 5 min seated rest in normoxia.

### 2.7 Gas exchange, ventilatory responses, operating lung volumes, and expiratory flow limitation

Gas exchange, ventilatory responses, operating lung volumes, and EFL were assessed via breath‐by‐breath analysis (Metalyzer 3B, Cortex, Leipzig, Germany) and spirometry software application (MetaSoft Studio, Cortex, Leipzig, Germany). All gas exchange and ventilatory variables (VO_2_, carbon dioxide production [VCO_2_], V_E_, f_B_, V_T_, deadspace ventilation, alveolar ventilation [V_A_], end‐tidal carbon dioxide [P_ET_CO_2_]) were averaged over 30 s at the end of each 5‐min segment. V_A_ was calculated via the following equation for the arterial partial pressure of CO_2_ (PaCO_2_):

$$P_{a}{\mathrm{CO}}_{2}={\left({\mathrm{VCO}}_{2}/V_{A}\right)}^{*}\;K.$$

P_ET_CO_2_ was assumed to be equal to P_a_CO_2_, and K is a conversion factor that adjusts V_A_ to body temperature and pressure. Deadspace ventilation was calculated as the difference between V_E_ and V_A_ (Stickland et al., 2013; West & Luks, 2020).

Carbohydrate (CHO) and fat oxidation (g·min^−1^) were calculated via application of stoichiochemical equations to VO_2_ and VCO_2_ on the assumption that protein oxidation is negligible during exercise (Jeukendrup & Wallis, 2005).

Operating lung volumes were estimated from IC maneuvers, which were completed at rest and every 5 min during exercise. End‐expiratory lung volume (EELV) was calculated by subtracting IC from forced vital capacity (FVC), and end‐inspiratory volume (EILV) was equal to V_T_ plus EELV. To determine EFL, tidal volume loops were assessed for overlap with the maximal flow‐volume loop. Incidences of overlap were measured to determine the severity with a minimum 5% being required to be categorized as EFL (Chapman et al., 1998). However, since emerging research has suggested that a higher threshold should be used for EFL presence when not correcting for thoracic gas compression, we also report EFL presence at a threshold of >40% (Strozza et al., 2020).

### 2.8 Maximal inspiratory and expiratory pressures

MIP and MEP were assessed at rest and within 4 min following exercise via a handheld pressure meter (MicroRPM Respiratory Pressure Meter, MD Spiro, Lewiston, ME, USA). For MIP, subjects were instructed to exhale to residual volume before maximally inhaling to total lung capacity. MEP was commenced at total lung capacity. Tests were repeated until there were two maximal values within 5 cmH_2_O.

### 2.9 Perceptual responses

Rating of perceived exertion and dyspnea were collected in the last 30 s of each 10‐min segment of exercise (i.e., starting at 5 min) using Borg scales (0–10) (Borg 1982).

### 2.10 Control procedures

All testing was completed following a 4 h fast and 24 h without exercise. All experimental trials were separated by at least 48 h. Subjects were asked to complete 24‐h and 72‐h diet and exercise logs, respectively, prior to each experimental trial. After the first trial, logs were copied and given back to subjects. For the final two trials, subjects were asked to maintain exercise consistency and replicate their diet for the 24 h preceding each experimental trial. Subjects were asked to abstain from caffeine and alcohol for 24 h and 48 h prior to experimental trials, respectively.

### 2.11 Statistical analysis

Means and standard deviations (SD) are presented for all dependent variables in text and tables. Means and standard errors of the mean (SE) are presented in figures to ease interpretation. All measurements assessed over time were analyzed via two‐way repeated measures analysis of variance (ANOVA) to identify main and interaction effects. Residuals produced by the ANOVA were assessed visually to confirm approximate normality, and a Greenhouse–Geisser correction was employed in the case that data violated sphericity. In the case of significant main effects or interactions, post hoc simple contrasts were utilized to identify differences between conditions at the different time points and within‐condition time effects. Pulmonary function, VO_2max_, MIP/MEP (change scores), and mean F_I_O_2_ were analyzed via one‐way repeated measures ANOVA with post hoc Bonferroni‐adjusted simple contrasts to identify significant differences. Time effects for MIP and MEP were analyzed via paired *t‐*test. Finally, for exploratory post hoc EFL analysis, exercise means were calculated for variables of interest and assessed via one‐way repeated measures ANOVA with EFL status included as a between‐subjects factor. In the case of a significant or trending (*p* < 0.1; i.e., to account for reduced statistical power) between‐subjects effects, further post hoc simple contrasts with Bonferroni correction were utilized to identify significant differences within each condition. All analyses were performed via IBM SPSS Statistics (Version 29). The *α* level for statistical significance was set at 0.05.

## 3 RESULTS

### 3.1 VO_2max_ , environmental conditions, and walking speeds

There was a reduction in VO_2max_ in hypoxia (37.2 ± 2.2 mL·kg^−1^·min^−1^) relative to normoxia (50.7 ± 4.2 mL·kg^−1^·min^−1^; *p* < 0.001). There were also differences between conditions for F_I_O_2_ (*p* < 0.001) and walking speed (*p* < 0.001). Specifically, F_I_O_2_ was reduced with UH (13.0 ± 0.03%) and LH (13.0 ± 0.04%) compared to UN (20.93 ± 0.001%), and the walking speed was slower with LH (4.3 ± 0.41 m·s^−1^) versus the other conditions (UN = 5.8 ± 0.4 m·s^−1^; UH = 5.8 ± 0.4 m·s^−1^; *p* < 0.001).

### 3.2 Pulmonary function

Pulmonary function results are presented in Table 1. There were differences between conditions for all pulmonary function variables (*p* < 0.01). Specifically, all pulmonary function variables, except for FEV_1_/FVC and FEV_1_/FVC % predicted, were reduced with LH relative to the other conditions (mean reductions of 8%, 10%, and 10% for FVC, FEV_1_, and PEF, respectively; *p* < 0.05). Additionally, FEV_1_/FVC and FEV_1_/FVC % predicted were increased with UH compared to UN (*p* = 0.007) and versus UN and LH (*p* < 0.05), respectively.

**TABLE 1:** Pulmonary function testing results.

	FVC (L)	FVC (%pred)	FEV_1_ (L)	FEV_1_ (%pred)	FEV_1_/FVC	FEV_1_/FVC (%pred)	PEF (L/s)	PEF (%pred)
UN	5.9 (0.7)	113.5 (13.9)	4.6 (0.5)	103.9 (13.9)	78.4 (5.4)	90.8 (5.9)	9.9 (1.4)	96.2 (13.8)
UH	5.8 (0.7)	112.8 (14.0)	4.6 (0.5)	105.5 (13.1)	80.0 (4.8)**	92.9 (5.5)*	9.7 (1.3)	93.3 (12.1)
LH	5.4 (0.6)*	104.0 (11.0)*	4.2 (0.5)*	95.1 (11.4)*	78.4 (5.0)	90.8 (5.9)	8.9 (1.6)*	85.4 (14.8)*

*Note*: Data are presented as mean (SD).

Abbreviations: %pred, percent of predicted; FEV1, forced expiratory volume in 1 s; FVC, forced vital capacity; PEF, peak expiratory flow.

* Denotes different from other conditions (*p* < 0.05).

** Denotes different versus UN (*p* < 0.05).

### 3.3 Ventilation

**FIGURE 2:**
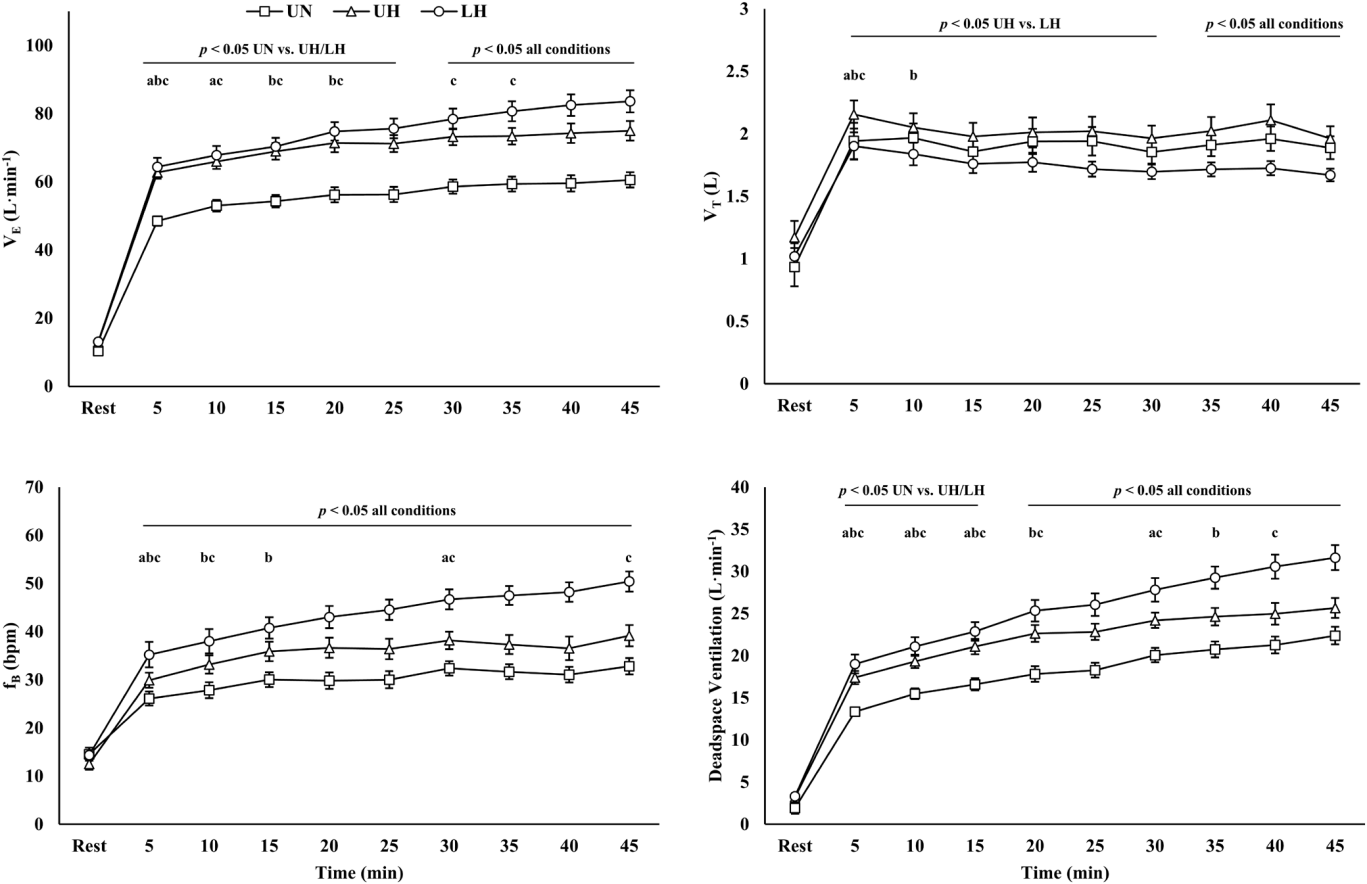
(a) Minute ventilation; (b) tidal volume; (c) breathing frequency; and (d) deadspace ventilation during exercise. Data are presented as mean ± SE. LH, loaded hypoxic; UH, unloaded hypoxic; UN, unloaded normoxic. ^a,b,c^Denotes different from prior time point within UN, UH, and LH, respectively (*p* < 0.05).

Ventilatory responses are presented in Figure 2 and Table 2. There were condition (*p* < 0.001), time (*p* < 0.001), and interaction effects (*p* < 0.001) for V_E_, f_B_, V_A_, deadspace ventilation, and P_ET_CO_2_. Additionally, there were condition and time effects for V_T_ (*p* < 0.001).

**TABLE 2:** Selected physiological and perceptual responses during exercise.

	UN			UH			LH		
	5 min	25 min	45 min	5 min	25 min	45 min	5 min	25 min	45 min
VO_2_ (L·min^−1^)	2.0 (0.1)^b^	2.1 (0.2)	2.2 (0.2)*	2.0 (0.2)	2.1 (0.2)*	2.2 (0.2)	1.9 (0.2)	2.1 (0.2)*	2.1 (0.2)
VCO_2_ (L·min^−1^)	1.7 (0.2)^c^	1.8 (0.2)	1.8 (0.2)	1.9 (0.2)	1.9 (0.2)	1.9 (0.2)	1.8 (0.1)	1.9 (0.1)	1.9 (0.1)
P_ET_CO_2_ (mmHg)	42.8 (3.5)^a^	42.1 (4.3)^a^	42.0 (4.0)^a^	35.5 (1.6)^b^	34.0 (1.8)*, ^b^	33.5 (1.8)^b^	34.0 (2.8)	32.4 (2.5)*	31.2 (2.5)*
V_A_ (L·min^−1^)	35.1 (3.6)^a^	38.0 (4.9)^a^	38.2 (4.5)^a^	45.3 (4.0)	48.4 (5.7)	49.3 (6.2)	45.3 (5.6)	49.6 (5.7)*	52.0 (6.4)
IC (L)	4.1 (0.5)	3.9 (0.6)	3.9 (0.5)^c^	3.9 (0.6)	3.9 (0.6)	3.7 (0.5)	3.8 (0.4)	3.5 (0.5)	3.5 (0.4)
SPO_2_ (%)	94 (3)^a^	94 (1)^a^	93 (2)^a^	74 (4)	73 (4)	75 (6)	75 (3)	74 (3)	75 (4)
SBP (mmHg)	166 (14)	166 (15)	158 (16)	173 (17)	171 (17)	173 (18)	161 (21)	170 (18)	169 (21)
DBP (mmHg)	56 (11)	54 (8)	51 (10)	55 (11)	48 (9)	45 (8)	71 (13)^a^	55 (10)	53 (12)
MAP (mmHg)	120 (37)	119 (37)	113 (35)	123 (6)	120 (38)	120 (38)	121 (39)	122 (38)	120 (39)
RPE (0–10)	1.1 (0.6)^b^	1.7 (0.9)*, ^a^	2.0 (1.0)^a^	1.7 (1.2)	2.7 (1.1)*, ^b^	3.3 (1.4)^b^	2.0 (1.0)	3.7 (1.0)*	4.8 (1.3)*
Dyspnea (0–10)	0.8 (0.8)^a^	1.8 (1.0)*, ^a^	2.0 (1.1)^a^	1.8 (1.1)	2.8 (1.1)*, ^b^	3.1 (1.4)^b^	2.0 (1.0)	3.5 (0.9)*	4.6 (1.2)*

*Note*: Data are presented as mean (SD).

Abbreviations: CHO, carbohydrate oxidation; DBP diastolic blood pressure; FAT, fat oxidation; IC, inspiratory capacity; P_ET_CO_2_, pressure of end‐tidal carbon dioxide; RPE, rating of perceived exertion; SBP, systolic blood pressure; SPO_2_, arterial oxygen saturation; V_A_, alveolar ventilation; VCO_2_, carbon dioxide production; VO_2_, oxygen consumption.

* Denotes different from lower time point.

^a^ Denotes different from other conditions (*p* < 0.05).

^b^ Denotes different from LH (*p* < 0.05).

^c^ Denotes different from UH (*p* < 0.05).

In general, hypoxia increased V_E_, f_B_, deadspace ventilation, and V_A_ during exercise (*p* < 0.05). For deadspace ventilation, V_E_, and f_B_ there were further elevations with LH compared to UH starting at 20 min, 30 min, and throughout exercise, respectively (*p* < 0.05). Conversely, P_ET_CO_2_ was decreased during exercise in the hypoxic conditions (*p* < 0.001), and the reduction was larger with LH versus UH (*p* < 0.05). V_T_ was reduced during exercise with LH compared to UH (*p* < 0.05), and there were differences between all conditions starting at 35 min (*p* < 0.05).

### 3.4 Operating lung volumes

**FIGURE 3:**
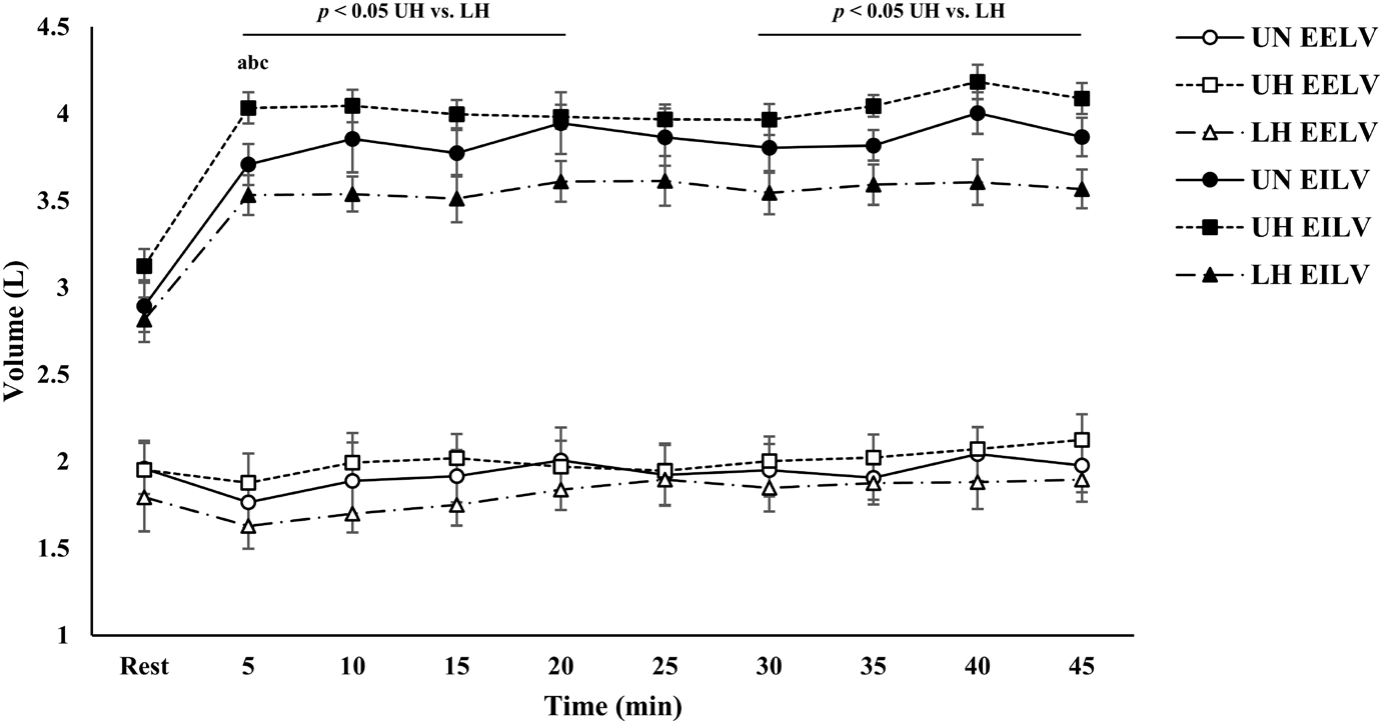
Operating lung volumes during exercise. Data are presented as mean ± SE. EELV, end expiratory lung volume; EILV, end inspiratory lung volume. ^a,b,c^Denotes different from prior time point within UN, UH, and LH, respectively (*p* < 0.05).

Operating lung volumes are presented in Figure 3. There were condition and time effects for absolute EILV (*p* < 0.01). Additionally, main effects for condition (*p* = 0.034) and time (*p* < 0.001) were present for IC and EELV (%FVC), respectively.

With LH, absolute EILV was reduced compared to UH during exercise (*p* < 0.05) with the exception of 25 min. There were no differences in EILV between LH and UN. Moreover, EELV was similar between conditions throughout exercise. For IC, there were reductions with LH (3.5 ± 0.4 L) versus UH (3.8 ± 0.5 L) at 20 min (*p* = 0.042). Additionally, IC was lower with LH (3.5 ± 0.5 L) relative to UN (3.9 ± 0.6 L) at 35 min (*p* = 0.022), and there was also a strong trend present at 45 min (*p* = 0.051). Finally, IC was reduced with UH versus UN at 45 min (*p* = 0.015).

EFL was observed in 6/12 subjects in the LH condition. Overall, the degree of encroachment on the maximal flow‐volume loop was substantial (mean ± SD: 58 ± 19%; range = 9–85.7%). In 4/6 subjects, EFL exceeded 40% overlap. For 3/4 of these subjects, EFL occurred at 5 min and persisted throughout exercise. Additionally, there was a trend for mean exercise absolute EELV to be different between conditions based on EFL status (*p* = 0.077). Post hoc analysis revealed that absolute EELV was lower among subjects with EFL in the LH condition (*p* = 0.022).

### 3.5 Maximal inspiratory and expiratory pressures

MIP and MEP are presented in Table 3. There were differences between conditions for MIP (*p* = 0.025) and MEP (*p* = 0.042). Specifically, there was a 12.2 ± 8.2% reduction in MIP with LH (*p* < 0.001), and this reduction was different from UN (*p* = 0.033). Notably, there was also a strong trend for a reduction in the degree of change in MIP with LH versus UH (*p* = 0.051). There were no differences in the degree of change between conditions for MEP. However, there were 4.1 ± 4.6% and 10.1 ± 9.5% reductions in MEP from pre‐ to post‐exercise with UH (*p* = 0.015) and LH (*p* = 0.005), respectively.

**TABLE 3:** Maximal inspiratory and expiratory pressure results.

	UN		UH		LH	
	Pre	Post	Pre	Post	Pre	Post
MIP (cmH_2_O)	134.2 (21.1)	136.5 (30.9)	133.0 (23.1)	142.3 (28.9)	135.0 (23.2)	118.5 (28.4)*, ^a^
MEP (cmH_2_O)	150.6 (22.2)	146.5 (19.0)	151.3 (18.5)	145.1 (14.7)*	154.2 (23.8)	138.5 (19.1)*

*Note*: Data are presented as mean (SD).

Abbreviations: LH, loaded hypoxic; MEP, maximal expiratory pressure; MIP, maximal inspiratory pressure; UH, unloaded hypoxic; UN, unloaded normoxic.

* Denotes different from pre‐exercise (*p* < 0.05).

^a^ Denotes a difference in the change between LH and UN (*p* < 0.05).

### 3.6 Hemodynamics

**FIGURE 4:**
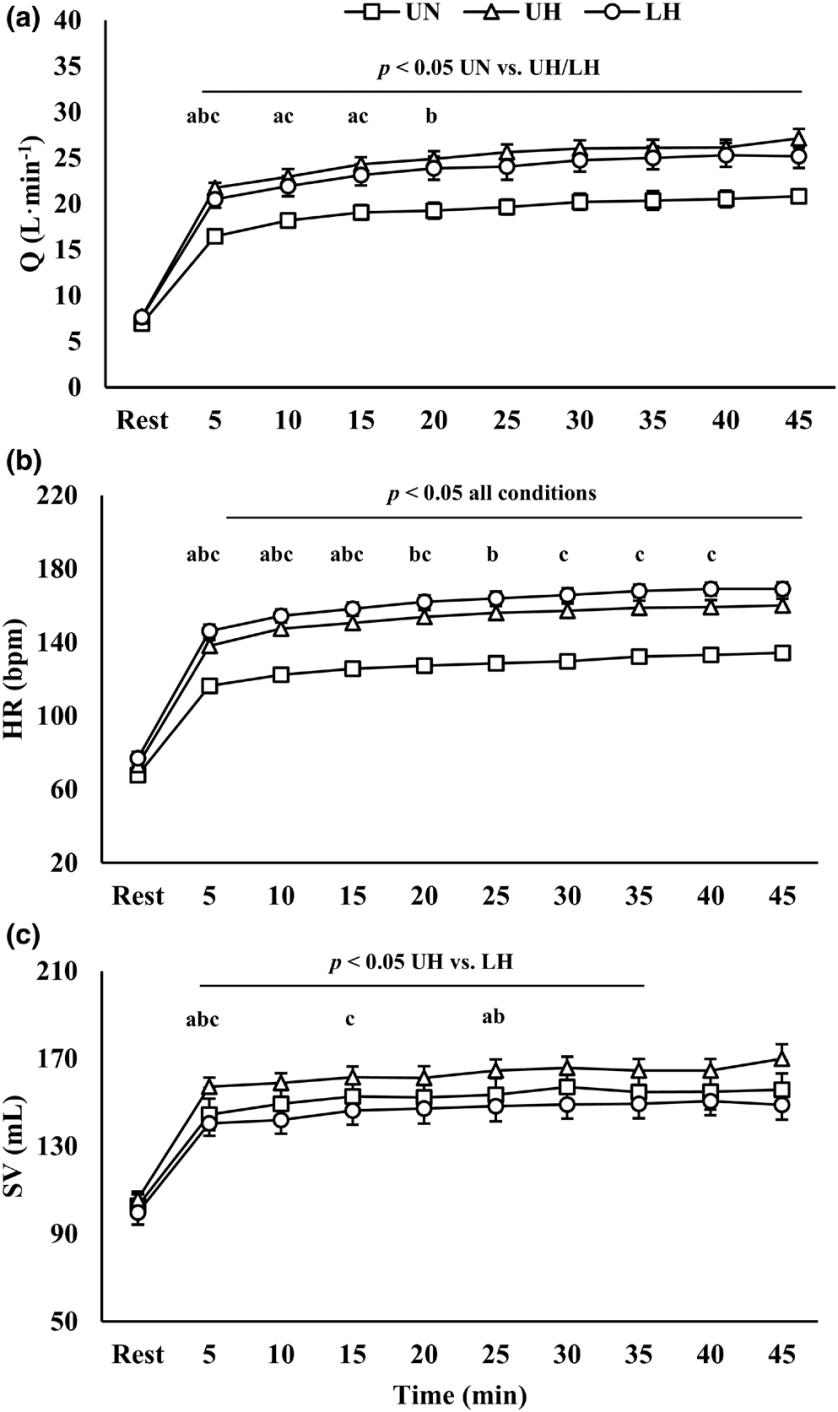
(a) Cardiac output; (b) heart rate; (c) stroke volume during exercise. Data are presented as mean ± SE. LH. Loaded hypoxic; UH, unloaded hypoxic; UN, unloaded normoxic. ^a,b,c^Denotes different from prior time point within UN, UH, and LH, respectively (*p* < 0.05).

Q, SV, HR, and SPO_2_ are presented in Figure 4 and Table 2. There were condition, time, and interaction effects for Q, HR, and SPO_2_ (*p* < 0.001). For SV, there were main effects for condition (*p* = 0.004) and time (*p* < 0.001), but no interaction effect was present.

In hypoxia, Q and HR were increased (*p* < 0.001). While there were no differences in Q between hypoxic conditions, HR was elevated further with LH compared to UH during exercise (*p* < 0.01). Additionally, SV was reduced with LH versus UH during exercise until 35 min (*p* < 0.05). There were no differences in SV between LH and UN throughout exercise. SPO_2_ was reduced in the hypoxic conditions (*p* < 0.001), but there were no differences between UH and LH.

For arterial blood pressure, there were time effects for SBP, DBP, and MAP (*p* ≤ 0.001). Moreover, there was a condition effect for DBP (*p* = 0.039). With LH, there was an increase in DBP at 5 min relative to UN (*p* = 0.032) and UH (*p* = 0.047). However, there were no other differences between conditions for any other timepoint or variable.

### 3.7 Muscle oxygen kinetics

**FIGURE 5:**
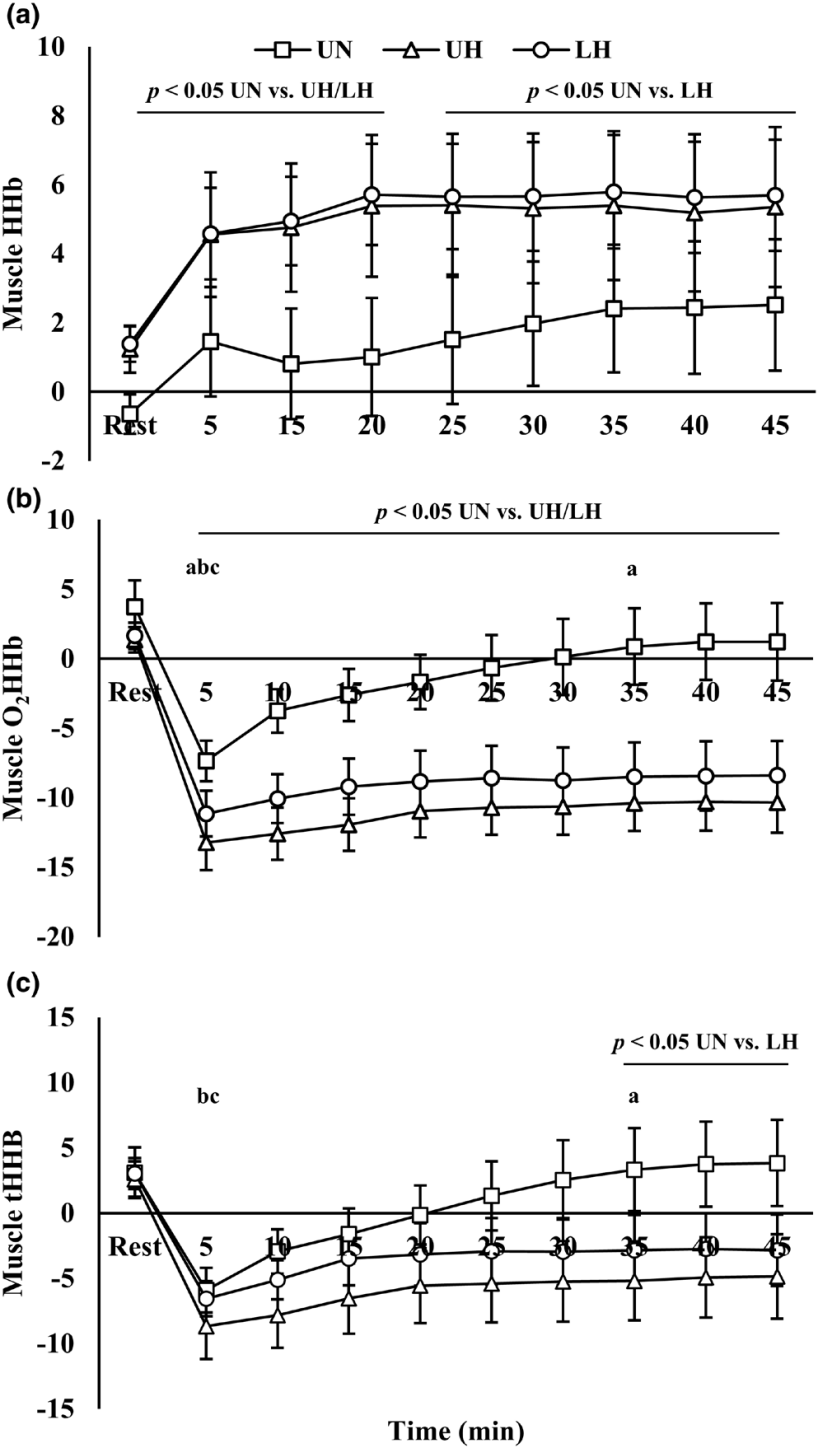
(a) Muscle deoxygenated hemoglobin; (b) muscle oxygenated hemoglobin; (c) muscle total hemoglobin. Data are presented as mean ± SE. LH, loaded hypoxic; UH, unloaded hypoxic; UN, unloaded normoxic. ^a,b,c^Denotes different from prior time point within UN, UH, and LH, respectively (*p* < 0.05).

Muscle oxygen kinetics are presented in Figure 5. There were condition, time, and interaction effects present for muscle O_2_HHb (*p* < 0.01). For muscle HHb and tHHb, there were main effects for condition and time (*p* < 0.05).

In hypoxia, muscle HHb and O_2_HHb were increased (*p* < 0.05) and decreased (*p* < 0.01), respectively, with no differences between UH and LH. For tHHb, there were no differences between UH and LH. However, tHHb was higher with UN compared to LH starting at 35 min (*p* < 0.05).

### 3.8 Cerebral oxygen kinetics

**FIGURE 6:**
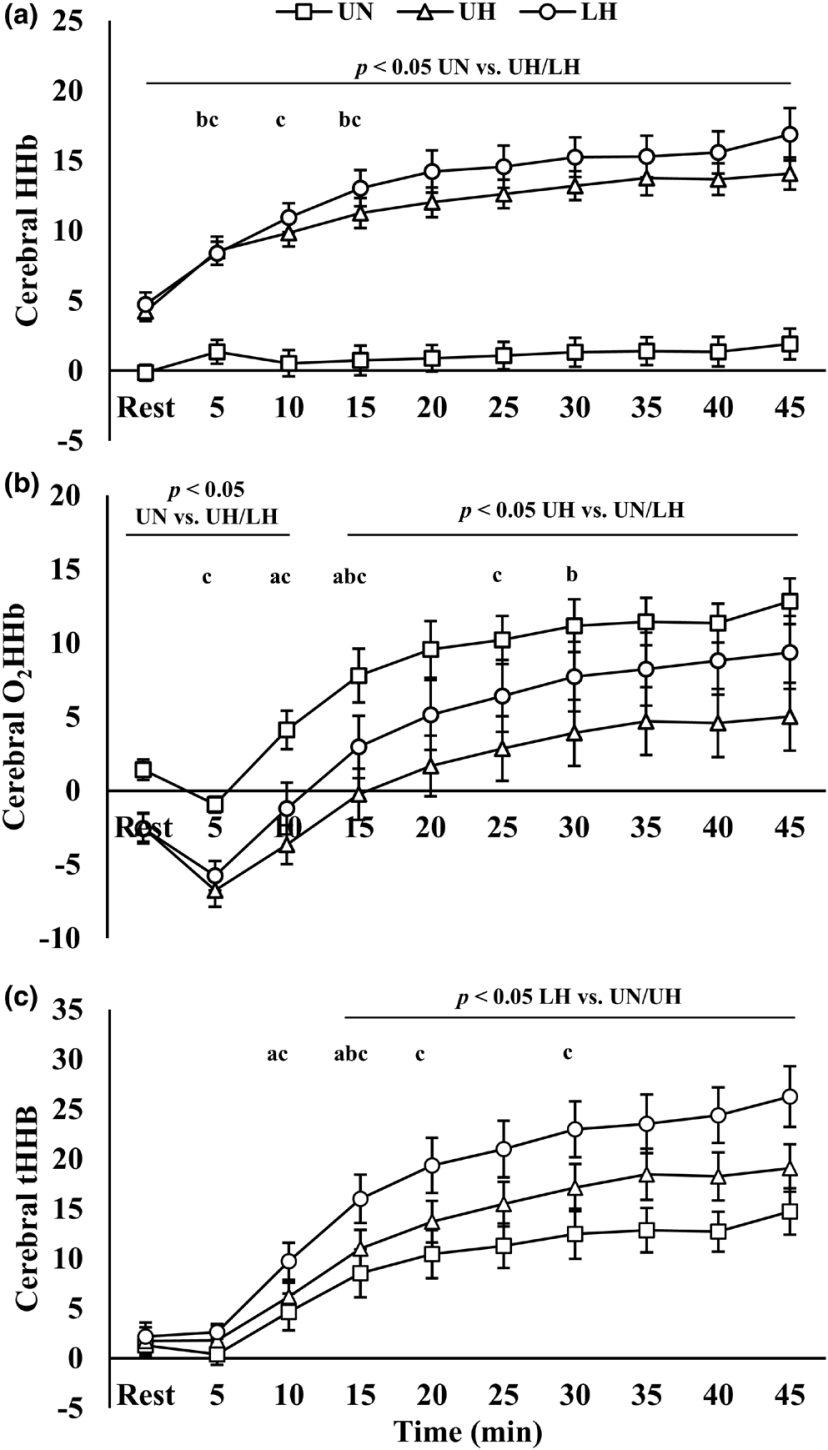
(a) Cerebral deoxygenated hemoglobin; (b) cerebral oxygenated hemoglobin; (c) cerebral total hemoglobin. Data are presented as mean ± SE. LH. loaded hypoxic; UH, unloaded hypoxic; UN, unloaded normoxic. ^a,b,c^Denotes different from prior time point within UN, UH, and LH, respectively (*p* < 0.05).

Cerebral oxygen kinetics are presented in Figure 6. There were condition, time, and interaction effects present for cerebral HHb and tHHb. For cerebral O_2_HHb, there were main effects for condition (*p* = 0.002) and time (*p* < 0.001).

Cerebral HHb was increased in hypoxia with no differences between UH and LH. Similarly, hypoxia reduced O_2_HHb until 10 min (*p* < 0.05). Thereafter, O_2_HHb was reduced only with UH (*p* < 0.05) while there were no differences between LH and UN. Starting at 15 min, tHHb was elevated with LH versus the other conditions (*p* < 0.05).

### 3.9 Gas exchange and perceptual responses

**FIGURE 7:**
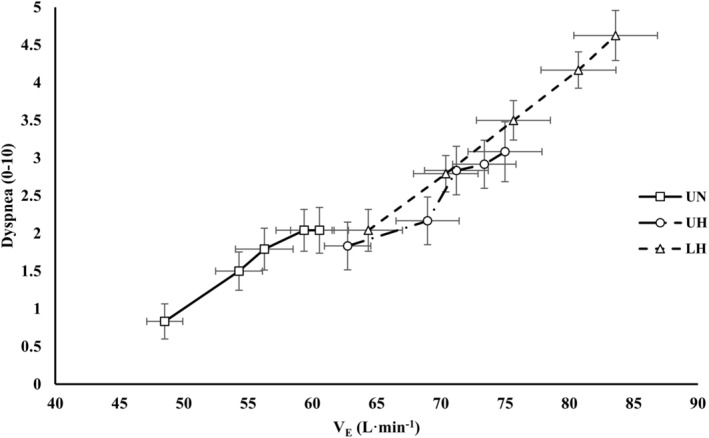
The relationship between dyspnea and minute ventilation. Data are presented as mean ± SE. LH, loaded hypoxic; UH, unloaded hypoxic; UN, unloaded normoxic; V_E_, minute ventilation.

Gas exchange and perceptual responses are presented in Table 2. The relationship between dyspnea and V_E_ is presented in Figure 7. For VCO_2_, there were main effects for condition (*p* = 0.048) and time (*p* < 0.001). There was a main effect of time for VO_2_ (*p* < 0.001).

For VO_2_ and VCO_2_, there were reductions and increases with LH versus UN (*p* = 0.001) and UH versus UN (*p* = 0.02) at 5 min, respectively. However, there were no other differences between conditions at any other time point.

There were condition, time, and interaction effects for RPE and dyspnea (*p* < 0.001). RPE and dyspnea were elevated during exercise in hypoxia (*p* < 0.05). Additionally, there were further elevations in RPE and dyspnea starting at 25 min with LH versus UH (*p* < 0.05).

## 4 DISCUSSION

This study investigated the physiological effects of prolonged thoracic load carriage in hypoxia. Primary findings from this study were as follows: (1) LH resulted in the immediate adoption of a shallow breathing pattern that increased deadspace ventilation and V_E_ leading to higher perceptions of exertion and dyspnea, (2) alterations in breathing patterns with LH were associated with inspiratory and expiratory muscle fatigue and EFL, (3) Q was maintained with LH versus UH despite a reduction in SV via a compensatory increase in HR, (4) arterial blood pressure and muscle oxygen kinetics were similar between hypoxic conditions suggesting the absence of a respiratory muscle metaboreflex, and (5) cerebral oxygenation and tHHb were increased with LH relative to UH indicating redistribution of blood to the pre‐frontal cortex.

### 4.1 Ventilatory responses

LH induced a shallow breathing pattern characterized by increases in f_B_ and reductions in V_T_ consequent to lower absolute EILV. As a result, deadspace ventilation was elevated starting at 20 min requiring compensatory increases in V_E_ to maintain V_A_ and hemoglobin saturation. These findings are similar to those reported with VO_2_‐matched prolonged load carriage in normoxia (Phillips, Stickland, & Petersen, 2016b). However, the timing of effects in the present study suggests that ventilatory responses may be distinct in hypoxia. Specifically, Phillips, Stickland, & Petersen (2016b) observed consistent ventilatory responses (i.e., V_E_, V_T_, and EILV) until 15 min of exercise (and which were not sustained until after 35 min). In contrast, breathing patterns were altered within 5 min of exercise (i.e., the first exercise measurement) in the present study. It is conceivable that these distinct findings are explained by differences in load mass (i.e., ~30 kg [present study] vs. 25 kg [Phillips et al.]). Alternatively, the explanation may be related to potential interactions between ventilatory demand, W_b_, and arterial oxygen content (CaO_2_). Specifically, it is likely that alterations in W_b_ are mediated by V_T_ and that with adaptive shallow breathing, the power of breathing (i.e., W_b_ × f_B_; joules·min^−1^) can be maintained relative to unloaded exercise (Dominelli et al., 2012). It is possible that this adaptive breathing pattern is mediated by CaO_2_ and more rapidly adopted in hypoxia, (where diaphragm fatigue occurs at lower intensities (Amann, Pegelow, et al., 2007)), in an effort to attenuate diaphragm VO_2_ and fatigue. Indeed, others have argued that diaphragm blood flow may be prioritized over locomotor muscles when there is competition for Q (Sheel et al., 2018), which provides some support for this theoretical diaphragm‐protective response. However, this is purely speculation. More research is needed to confirm this as most prior studies evaluating ventilatory responses to load carriage or resistive breathing in hypoxia have either employed non‐VO_2_‐matched designs (Hinde et al., 2018) or controlled breathing patterns, which prevent detection of adaptive breathing (Katayama et al., 2013b; Verges et al., 2010).

As noted, LH resulted in reductions in absolute EILV, but EELV did not change. Most prior load carriage research supports this finding (Phillips et al., 2019; Phillips, Stickland, & Petersen, 2016b, 2016c) and was expected given the above‐discussed adaptive breathing pattern adopted with thoracic load carriage. However, others have reported reductions in EELV combined with consistent EILV (Dominelli et al., 2012) or increases in both EELV and EILV (Armstrong et al., 2019) with progressively increasing loads. It is possible that discrepancies in operating lung volume results are a consequence of differences in study design as both Dominelli et al. (2012) and Armstrong et al. (2019) employed consistent speed and grade across all loads resulting in higher work rates and presumably requiring larger V_T_ (i.e., which would be achieved via decreases in EELV in order to maintain the diaphragm at optimal length (Aliverti, 2008) in the loaded conditions). Moreover, Armstrong et al. (2019) combined pack load carriage with body armor, which independently increases chest wall restriction (Armstrong & Gay, 2016). Thus, more research with consistent study designs is needed to determine the precise impact of load carriage on operating lung volumes and whether effects are mediated by hypoxia. A unique finding in the present study relative to prior load carriage (Armstrong et al., 2019; Phillips et al., 2019; Phillips, Ehnes, et al., 2016; Phillips, Stickland, & Petersen, 2016c) and chest wall restriction (Tomczak et al., 2011) studies was that EILV presented as a percentage of FVC was not changed. Rather, changes in operating lung volumes seemed to mirror changes in FVC such that subjects effectively reproduced their normal breathing pattern at a smaller scale. An explanation for this is not immediately clear but may be a consequence of increased EELV variability between subjects given selective EFL presence and potential hyperinflation (discussed below). Regardless, when operating lung volume results from the present study are viewed in light of the fact that load carriage does not reduce total lung capacity (Phillips, Stickland, & Petersen, 2016b), changes in absolute volumes are accurately reflective of load carriage effects on the utilization of available lung capacity.

Reductions in MIP and MEP with LH indicated respiratory muscle fatigue. Our finding of reduced MIP is consistent with prior studies in normoxia (Faghy & Brown, 2014; Phillips, Stickland, & Petersen, 2016b, 2016c) and hypoxia (Hinde et al., 2018) and can likely be explained by alterations in operating lung volumes induced by chest wall restriction, which increase W_b_ (Brown & McConnell, 2012; Faghy et al., 2022). Interestingly, the magnitude reduction observed in the present study (12%) exceeds that reported previously with normoxic load carriage (7%) given a similar load (25 vs. 29.5 kg), duration (45 min), and relative intensity (65 %VO_2peak_) of exercise (Phillips, Stickland, & Petersen, 2016b). It is possible that this is evidence of increased respiratory muscle fatiguability in hypoxia (Verges et al., 2010), a notion also supported by the fact that work rates (VO_2_ = ~2.0 vs. 3.0 L·min^−1^) and V_E_ (~60–85 vs. 75–100 L·min^−1^) were lower in the present study. Nevertheless, others have reported similar reductions in MIP (11%) with prolonged normoxic load carriage in males (Faghy & Brown, 2014) and females (Phillips, Stickland, & Petersen, 2016c). While this may be explained by methodological differences (e.g., females carrying a higher relative load, longer exercise duration, etc.), more research is needed to clarify if hypoxia magnifies load carriage‐induced respiratory muscle fatigue. Also worth noting, there were time effects for MEP for both UH (4%) and LH (10%). Changes in MEP with thoracic load carriage have been reported by some in normoxia (Armstrong et al., 2019; Faghy & Brown, 2014) and hypoxia (Hinde et al., 2018), but not others (Phillips, Stickland, & Petersen, 2016b, 2016c). Evidence for fatigue of both inspiratory and expiratory muscles could suggest centrally‐mediated whole‐body reductions in motor output, which has been observed with increasing levels of hypoxia (Amann, Romer, et al., 2007). However, the absence of change in MIP with UH would suggest that peripheral fatigue of both the inspiratory and expiratory muscles is the more likely mechanism with LH. Regardless, more research potentially employing comparisons between MIP/MEP and bilateral phrenic nerve stimulation following hypoxic load carriage exercise is needed to determine the degree to which respiratory muscle fatigue is centrally or peripherally mediated.

A novel finding from the present study was the presence of EFL in 50% of subjects in the LH condition. Moreover, EFL exceeded 40% overlap with the maximal flow‐volume loop in 4/6 subjects and occurred early in exercise (5–25 min) under relatively modest ventilatory demands (60–75 L·min^−1^). These findings were noteworthy, particularly given that EFL with non‐loaded exercise typically occurs at V_E_ rates >120 L·min^−1^ in trained endurance athletes (Johnson et al., 1992; McClaran et al., 1999). Similar incidence rates (≤11/15) and severity of EFL (~25%–72%) have been reported previously in normoxia with progressively increasingly loads and work rates (Armstrong et al., 2019). However, this study combined pack load carriage with body armor resulting in heavy total loads (~27–47 kg) and increased chest wall restriction that reduced FVC to a larger extent (even at comparable load) compared to the present study (12%–15% vs. 8%). These conditions likely exacerbated flow limitation. Indeed, a study employing inelastic chest strapping (Tomczak et al., 2011) that induced severe restriction (>40% reduction in FVC) reported EFL in 100% of participants at similar ventilatory demands compared to Armstrong et al. (Tomczak et al., 2011). In a study employing loaded conditions more comparable to the present study (i.e., 15–35 kg pack loads), relatively minor EFL (7%–12% severity) was reported in a small number of subjects (2/7) and only under the heaviest load (35 kg; (Duncan et al., 1995)). It is possible that differences in incidence rates and severity are explained by the fact that this study accounted for thoracic gas compression, which was not done in the present study. Alternatively, it is tempting to interpret the present EFL findings as being hypoxia‐specific, perhaps indicating a distinct alteration in operating lung volumes in order to minimize changes in W_b_ or dyspnea. In support, EFL subjects in the present study had lower EELV compared to non‐EFL subjects, which would have limited expiratory flow capacity (and contributed to EFL) but permitted the diaphragm to operate at a more optimal length‐tension (Aliverti, 2008; Johnson et al., 1999). It is possible that this breathing strategy was prioritized to a larger degree in hypoxia to maximize diaphragm efficiency and endurance. If so, similar levels of dyspnea and ∆MIP between EFL and non‐EFL subjects suggest this was an effective response, at least given the ventilatory demands in the present study. More research is clearly needed that employs normoxic versus hypoxic comparisons between loaded and unloaded conditions to confirm this hypothetical mechanism.

### 4.2 Hemodynamics and oxygen kinetics

With thoracic load carriage in hypoxia, cardiovascular efficiency was impaired based on a reduction in SV (until 40 min of exercise) and increase in HR to achieve a similar Q to the UH condition. This has been reported previously with inelastic chest strapping (Miller et al., 2002) and normoxic load carriage (Baur et al., 2023). However, this is the first study to observe load carriage‐induced reductions in SV in hypoxia. This finding is likely explained by a reduction in end diastolic volume caused by attenuated negative pressure swings during inspiration resulting from chest wall restriction (Baur et al., 2023; Miller et al., 2005). It is also possible that reductions in SV were influenced by EFL, which can independently reduce SV due to the increases in intrathoracic pressure generated during expiration that increase right atrial pressure and reduce venous return (Stark‐Leyva et al., 2004). However, we did not observe any differences in SV between EFL and non‐EFL subjects suggesting this mechanism had limited or null effect. Regardless of the precise mechanism, this finding suggests cardiovascular limitations with load carriage similar to those observed with chronic heart failure and chronic obstructive pulmonary disease that could reduce VO_2max_ and hasten fatigue development via reductions in maximal Q and increases in vascular resistance stemming from an elevated pressor response (Amann et al., 2010; Smith et al., 2020). Furthermore, these responses may be exacerbated by a potential respiratory muscle metaboreflex (discussed below) induced by resistive breathing (Harms et al., 1997). The combination of these effects may explain previously observed reductions in VO_2max_/VO_2peak_ (Baur et al., 2023; Phillips et al., 2019; Phillips, Stickland, & Petersen, 2016b, 2016c) and impaired time trial performance (Faghy & Brown, 2014) with load carriage. Nevertheless, the available evidence suggests that cardiovascular inefficiency during submaximal exercise may not be sufficiently limiting to result in an exaggerated pressor response. For instance, Baur et al. (2023) reported similar ejection fractions and decreases in systemic vascular resistance compared to VO_2_‐matched normoxic unloaded and loaded walking. Meanwhile, the present study observed similar arterial blood pressures, which suggests consistent levels of sympathetic activity between conditions. Perhaps future studies employing increased hypoxia, longer duration exercise, or higher‐intensities may challenge Q sufficiently to clarify the mechanism and consequences of load carriage‐induced hemodynamic impairments. In support of this hypothesis, differences in SV between hypoxic conditions were nullified at 40–45 min possibly indicating the onset of a peripheral fatigue‐induced increase in sympathetic activity that may have been magnified give a longer exercise duration.

Muscle oxygenation was similar between hypoxic conditions. Specifically, O_2_HHb and HHb were reduced and increased, respectively, during exercise in hypoxia relative to normoxia. This finding is supported by others (Rosales et al., 2022; Subudhi et al., 2007, 2008) and is logical given the lower oxygen availability, which would require increased oxygen extraction from hemoglobin to maintain VO_2_. Nevertheless, this finding was somewhat surprising given that load carriage increases recruitment of more inefficient Type II fibers (Simpson et al., 2011) and accessory muscles in the shoulders, back, and abdomen that may conceivably alter region/muscle‐specific VO_2_ or the distribution of blood (Devroey et al., 2007). Moreover, Baur et al. (2023) observed reductions in muscle oxygenation with speed‐matched hypoxic load carriage relative to unloaded normoxic walking. However, this study did not have an unloaded hypoxic comparison condition increasing the likelihood that this response was primarily the result of increased oxygen demand to maintain the same walking speed while carrying the load combined with the relative lack of oxygen. As with results for hemodynamics, it seems likely that the load carriage exercise challenge may have been insufficient to require substantial alterations in oxygen extraction or blood flow redistribution.

Given the similarities in muscle oxygen kinetics and arterial blood pressures, hypoxic load carriage did not appear to induce a respiratory muscle metaboreflex. Prior research has established that increased respiratory muscle work can result in a metaboreflex that reduces locomotor muscle blood flow and increases peripheral fatigue at high exercise intensities (>85% VO_2max_; (Hinde et al., 2018)). While this effect is not observed at lower submaximal intensities (Katayama et al., 2019), it was conceivable that load carriage‐induced increases in W_b_ combined with the increases in sympathetic activity (Katayama et al., 2013b) and respiratory muscle fatiguability (Verges et al., 2010) imposed by hypoxia may have been sufficient to cause blood flow redistribution even at the moderate work rate (2.0 L·min^−1^) and relative intensity (65% VO_2max_) employed in this study. The present results suggest that even under these conditions and with 10%–12% reductions in MIP/MEP indicating respiratory muscle fatigue, Q is not sufficiently limiting to require detectable alterations in muscle blood flow/oxygenation. While it must be acknowledged that NIRs does not provide direct measurement of blood flow and that subtle changes in flow or vascular conductance may have occurred (Katayama et al., 2019), it seems likely that any meaningful metaboreflex response influencing oxygen delivery would be indirectly evidenced via alterations in oxygen kinetics, which were also similar between hypoxic conditions. With that in mind, the combined ventilatory and locomotor work of thoracic load carriage do not appear to create a competition for Q even in hypoxia at intensities associated with time‐sensitive emergency response maneuvers (Ruby et al., 2003). Nevertheless, it is possible that a metaboreflex‐induced reduction in locomotor blood flow may be more apparent in females who have a higher W_b_ during exercise due to smaller airways (Dominelli et al., 2015), but this may be counterbalanced because females have greater resistance to respiratory muscle fatigue and a decreased metaboreflex response (Leahy et al., 2024). More research evaluating sex‐specific effects of load carriage is clearly warranted, particularly since women now serve in combat arms. Additionally, the effects of pre‐fatiguing the respiratory muscles, (which would occur with a prolonged submaximal load carriage task), on subsequent unloaded high‐intensity (>85% VO_2max_) exercise responses, (as might occur with evasive maneuvers following warfighter enemy contact or escape to safety for wildland firefighters), have not been investigated. This realistic scenario may reveal important practical implications of load carriage‐induced respiratory muscle fatigue and also warrants further study.

Interestingly, cerebral O_2_HHb and tHHb were elevated with LH compared to UH. This finding confirms a recent study of normoxic and hypoxic load carriage (Baur et al., 2023) and is suggestive of enhanced blood flow to the prefrontal cortex. This response is surprising given the typically close relationship between the partial pressure of CO_2_ and cerebral blood flow (Ogoh & Ainslie, 2009). We observed reductions in P_ET_CO_2_ with hypoxic load carriage due to hyperventilation (i.e., owing to increased f_B_ and deadspace ventilation), that would be expected to decrease cerebral blood flow. However, it is possible that changes in cerebral oxygen kinetics are primarily reflective of alterations in blood distribution within the brain rather than flow. This seems most likely given the similarities in Q and muscle oxygen kinetics between hypoxic conditions. Moreover, others have reported discrepancies between O_2_HHb/tHHb and cerebral blood flow (assessed via middle cerebral artery velocity [MCAv]) that support this potential mechanism. For instance, Subudhi et al. (Subudhi et al., 2008) observed an inverse relationship between tHHb and MCAv at high exercise intensities in normoxia and hypoxia. Moreover, similar to the present study, Bhambhani et al. (2014) reported an increase in O_2_HHb/tHHb during high relative to low intensity exercise (i.e., repeated isometric biceps brachii contractions), but this occurred despite similar MCAv across intensities. While the mechanism requires more research, cerebral blood flow redistribution to the prefrontal cortex is logical given the unique combination of factors potentially present during load carriage. Specifically, there is likely to be a conflict between hyperventilation‐induced cerebral vasoconstriction and the increased neuronal activation/metabolic demand in the prefrontal cortex stemming from balancing/carrying the load. This may result in region‐specific hypoperfusion that results in parasympathetic‐mediated vasodilation that would appear as an increase in tHHb (Kano et al., 1991). Importantly, this purported mechanism is likely to be exacerbated in hypoxia where oxygenation is further reduced and cerebral autoregulation is impaired (Ainslie et al., 2007; Bourdillon et al., 2014; Rosales et al., 2022). If true, future studies should evaluate whether these effects influence rates/severity of altitude illness or cognitive function given the potential for differential levels of perfusion throughout the brain.

### 4.3 Oxygen consumption and perceptual responses

VO_2_ increased during exercise, but there were no differences in VO_2_ drift between conditions. This finding was unexpected as several prior studies have reported VO_2_ drift with prolonged normoxic load carriage (Blacker et al., 2009, 2011; Lidstone et al., 2017; Patton et al., 1991; Phillips, Stickland, & Petersen, 2016b). Moreover, a review of load carriage research (Boffey et al., 2019) statistically calculated 47% VO_2max_ to be the threshold for the maintenance of cardiovascular efficiency, which was significantly exceeded in the present study. One possible explanation is that most studies employed matched speed designs (e.g., either non‐VO_2_‐matched, or VO_2_‐matched via changes in gradient) requiring relatively higher and matched walking cadences. However, cycling and running cadences do not appear to influence VO_2_ drift (Billat et al., 1999). Alternatively, the work rate (Phillips, Stickland, & Petersen, 2016b), exercise duration (Blacker et al., 2009, 2011), and relative load (Lidstone et al., 2017) was less in the present study relative to others, which may have attenuated fatigue or muscle damage‐induced decreases in efficiency. Indeed, no differences in VO_2_ drift were observed among females in a normoxic load carriage task similar to the present study (25 kg; 45 min; VO_2_: 2.0 L·min^−1^; (Richalet, 2020)).

Finally, perceptual responses indicated that hypoxic load carriage increased exertion and dyspnea following 25 min or exercise. This aligns closely with prior studies employing similar loads (25 kg) and exercise durations (45 min) in normoxia (Phillips, Stickland, & Petersen, 2016b, 2016c). Additionally similar to others (Phillips, Stickland, & Petersen, 2016b), load carriage‐induced increases in dyspnea seemed to closely correlate with V_E_ (see Figure 7). Differences in V_E_, may also partially explain alterations in perceived exertion as f_B_ has been shown to strongly predict perceptions of effort (Nicolò et al., 2016); although distinct muscular loading demands, accessory muscle recruitment, and increased respiratory work likely also contributed.

### 4.4 Limitations and conclusions

This study had several limitations. First, there is a growing body of research demonstrating that physiological responses to normobaric hypoxia may differ from hypobaric hypoxia (Angeli et al., 2019; Beidleman et al., 2014; Loeppky et al., 1997; Rosales et al., 2022; Woods et al., 2017). While this is still controversial (Richalet, 2020), it is possible that responses observed in this study are not accurately reflective of those expected in hypobaric hypoxia or actual altitude. Indeed, it is possible that ventilatory responses and/or W_b_ in hypobaric hypoxia may be distinct from normobaric hypoxia given the lower air density (Loeppky et al., 1997). Nevertheless, some well‐controlled studies comparing effects between conditions have reported no differences (Woods et al., 2017) or exacerbated responses to hypobaric hypoxia relative to normobaric hypoxia (Beidleman et al., 2014; Rosales et al., 2022). As such, is possible that our observations may understate effects in actual altitude making the observed magnitudes in this study all the more striking and potentially impactful. Additionally, our study design did not permit control for thoracic gas compression. As such, EFL incidences and severity may have been inflated. However, given that 4/6 subjects experienced >40% EFL, it seems likely that controlling for thoracic gas compression may have attenuated severity results, but not substantially impacted incidence rates. Finally, chest strap tension was not controlled in this study owing to differences in body dimensions between subjects. This may have resulted in differences in chest wall restriction that could have influenced results. Future studies should control for this variable and generally aim to differentiate between the effects of mass load and chest wall restriction. This question is highly relevant to tactical populations given the various forms of load carriage and protective equipment worn (e.g., body armor, backpacks, etc.), which may have distinctive physiological effects.

In conclusion, hypoxic thoracic load carriage resulted in reductions in cardiopulmonary efficiency that were associated with respiratory muscle fatigue and EFL. The time course of changes in breathing patterns and the incidence/severity of EFL indicated exacerbated effects in hypoxia relative to prior normoxic studies. Nevertheless, there was limited evidence for a respiratory muscle metaboreflex sufficient to influence locomotor muscle oxygenation. In addition, we confirmed prior evidence (Baur et al., 2023) showing increases in cerebral O_2_HHb and tHHb with load carriage that indicate redistribution of blood to the prefrontal cortex. In general, these physiological responses indicate substantial increases in physiological strain that interfere with the compensatory physiological response to hypoxia.

## CONFLICT OF INTEREST STATEMENT

The authors report no conflicts of interest.

## Data Availability

The data that support the findings of this study are available from the corresponding author upon reasonable request.
